# The Role of Artificial Intelligence and Machine Learning Amid the COVID-19 Pandemic: What Lessons Are We Learning on 4IR and the Sustainable Development Goals

**DOI:** 10.3390/ijerph19031879

**Published:** 2022-02-08

**Authors:** David Mhlanga

**Affiliations:** Faculty of Business and Economics, University of Johannesburg, Johannesburg 2006, South Africa; dmhlanga@uj.ac.za or dmhlanga67@gmail.com

**Keywords:** artificial intelligence, fourth industrial revolution lessons, sustainable development goals

## Abstract

The COVID-19 pandemic came with disruptions in every aspect of human existence, with all the sectors of the economies of the world affected greatly. In the health sector, the pandemic halted and reversed progress in health and subsequently shortened life expectancy, especially in developing and underdeveloped nations. On the other hand, machine learning and artificial intelligence contributed a great deal to the handling of the pandemic globally. Therefore, the current study aimed to assess the role played by artificial intelligence and machine learning in addressing the dangers posed by the COVID-19 pandemic, as well as extrapolate the lessons on the fourth industrial revolution and sustainable development goals. Using qualitative content analysis, the results indicated that artificial intelligence and machine learning played an important role in the response to the challenges posed by the COVID-19 pandemic. Artificial intelligence, machine learning, and various digital communication tools through telehealth performed meaningful roles in scaling customer communications, provided a platform for understanding how COVID-19 spreads, and sped up research and treatment of COVID-19, among other notable achievements. The lessons we draw from this is that, despite the disruptions and the rise in the number of unintended consequences of technology in the fourth industrial revolution, the role played by artificial intelligence and machine learning motivates us to conclude that governments must build trust in these technologies, to address health problems going forward, to ensure that the sustainable development goals related to good health and wellbeing are achieved.

## 1. Introduction

The COVID-19 pandemic affected the lives of the people and the economies around the globe. The psychosocial environment was changed drastically due to isolation, economic shutdowns, social distancing, among other restrictions, and these changes seriously affected countries [1]. Children, adolescents, and families were seriously affected. Leisure activities were limited, Schools and Kindergartens were closed, and social contacts were also limited due to social distancing. On the other hand, Parents were burdened with work, supporting children with schoolwork, and many were working from home. Apart from those challenges related to the economic meltdown, unemployment affected people’s mental health in a great way [1]. Razu et al. [1] also pointed out that the pandemic had a huge burden on healthcare professionals who were involved in curing the COVID-19 patients. All the healthcare professionals were at huge risk of contracting the virus, and, as a result, the psyche of healthcare workers was affected, coupled with professional stress, fear of infection, and even feeling helpless [2]. Fagherazzi et al. [3] also highlighted that the biggest impact of the virus was on the healthcare systems of the countries around the globe, as they were supposed to adapt to the increase in demand over the shortest period. Apart from that, there was the need for a serious coordinated approach to responding to the pandemic. Apart from that, COVID-19 was a unique disease because of the number of people infected, the rate of transmission, and the spectrum of clinical severity. As a result, the COVID-19 pandemic had a greater impact than previous pandemics such as influenza, severe acute respiratory syndrome (SARS), Ebola virus, and the Middle East respiratory syndrome (MERS). According to Fagherazzi et al. [3], the COVID-19 pandemic was the first true global pandemic in the digital era, giving digital health solutions a chance. Fagherazzi et al. [3] believe that digital health solutions had reached a certain level of maturity, but they were not widely deployed or accepted yet in the sector, and they played a major role in responding to the crisis. In a way, the pandemic provided the platform for health practitioners to start thinking about how digital health solutions can and should be leveraged to combat the crisis. Haleem and Javaid [4] posit that medical 4.0 was one of the key tools that could help in addressing the pandemic. Haleem and Javaid [4] highlight that medical 4.0 has some applications of advanced technologies, which can assist in addressing the challenges of the COVID-19 pandemic. As a result, the application of artificial intelligence (AI) and machine learning to COVID-19 research has been increasing. Islam et al. [5] posit that AI has been applied to COVID-19 research in areas related to diagnosis, classification, detection, severity, and mortality risk. Islam et al. [5] further argued that, even before the pandemic, the application of AI and machine learning has been on the rise. Sophisticated AI algorithms have been developed to solve complex tasks efficiently and effectively [6]. Amid the COVID-19 pandemic, AI became one of the serious tools that were used in monitoring and controlling the spread of the COVID-19 virus. Senthilraja, [6] stated that medical professionals began to look for technologies to monitor and control the pandemic. On the other hand, Harrus and Wyndham [7] highlighted that those technologies always come forth with intended and unintended consequences. However, the potential of AI has been so visible in its application in combating the negative implications of the pandemic that affected the world. The number of applications for AI exploded the moment the pandemic emerged in China.

According to Harrus and Wyndham [7], the COVID-19 pandemic acted as a catalyst for the saving or redemption of AI practitioners. The pandemic presented a unique opportunity to prove that AI can be harnessed to benefit humanity. However, the problems associated with the use of technology persisted just as the pandemic itself. Harrus and Wyndham [7] indicated that the use of AI in the fight against COVID-19, in a way, exacerbated the existing issues impacting the poor. Social inequalities across the world were exacerbated by the COVID-19 pandemic. The use of technology such as AI did little to address the disparities that existed before the pandemic lack of access to healthcare, resource inequalities, and disparities in the quality of care received did not improve significantly, especially for the vulnerable communities of society because of various issues such as lack of data and challenges related to the universal acceptance for telemedicine and many other reasons. For instance, the data produced by The American Public Media Research Lab indicated that cashiers, cleaning crews, delivery services, restaurant servers, and trade workers and their families were exposed to the virus in higher proportions compared to other groups in other occupations. Other groups that were highly exposed are the healthcare professionals, teachers, and nursing home workers, among others. Despite the misgivings and the negative implications of the pandemic on humanity, Senthilraja [6] indicated that AI played a critical role in tracking the spread of the virus, the identification of high-risk patients, and controlling the pandemic in real-time. Senthilraja [6] went on to state that AI also helped in predicting the mortality risk through effective analysis of previous data of the patients.

The other important issue that Senthilraja [6] outlined was that AI helped in the fight against COVID-19 through patient screening, medical check-ups, and assistance in notifying and suggesting infection control. Again, AI helped in improving the planning and treatment of COVID-19 patients [6]. Islam et al. [5] also argued that the application of AI to address the impact of the COVID-19 pandemic increased, especially, on diagnosis, classification, detection, severity, and mortality risk. Another study by Vaishya et al. [8] argued that healthcare, in general, requires maximum support from the new advanced technologies, such as AI internet of things, big data, and machine learning. Through the review of literature Vaishya et al. [8] discovered that there were a lot of applications that were applied in detecting clusters of COVID-19 cases and the prediction of where the virus will affect in the future through collecting and analysing the historical data. Vaishya et al. [8] concluded that decision-making technologies such as AI are critical in the handling of viruses and very useful in the development of vaccines. Another study by Khan et al., [9] also highlighted that AI has been successfully employed in combating the negative implications of the deadly COVID-19 pandemic. Khan et al. [9] discovered that AI has been used for the detection of the COVID-19 pandemic, screening, classification, drug repurposing, prediction of the virus, and forecasting. It is against this background that the current research seeks to investigate the role of AI and machine learning in addressing the COVID-19 Pandemic and to come up with lessons we are learning on the fourth industrial revolution and the sustainable development goals, with much focus on goal three.

## 2. Sustainable Development Goals (SDGs)

The SDGs are generally argued to be integrated, indivisible, and they are global [10]. The other features of the SDGs are that these goals are universally applicable, they can be applied in different countries with unique realities, capabilities, and levels of development [11]. The United Nations [10] also stated that the SDGs generally respect the national policies and priorities in various countries across the globe. On the other hand, the targets that follow the different SDGs are viewed by the United Nations [10] as aspirational and global, and each country sets its national targets considering the national circumstances and is guided by the global level of ambition. The other important aspect of the targets is that each government decide on their own how each of the global targets should be incorporated into national planning processes, policies, and strategies. When the United Nations and other stakeholders came forth with the goals and targets, they took into consideration the special challenges facing the most vulnerable nations, especially African countries, the least developed nations, the landlocked developing nations, as well as Small Island developing states. The development of the goals also took into consideration the specific challenges facing the middle-income countries as well as the circumstances around the countries in situations of conflict.

One of the major constraints for the effective monitoring and evaluation of progress and performance of the member states, in line with the SDGs, is the widespread lack of baseline data on many targets. The developments of the fourth industrial revolution present opportunities for effective data mining to ensure that national and global baselines become available, making it possible to measure the progress of the member states, especially on targets without numerical and clear targets. Table 1 below is summarizing the seventeen sustainable development goals.

Table one above summarizes the seventeen sustainable development goals. Even though the COVID-19 pandemic affected all the goals from one to seventeen, goal three was seriously affected. Goal three seeks to ensure health and well-being for all, including a bold commitment to end epidemics of AIDS, tuberculosis, malaria, and other communicable diseases by 2030 [11,12]. The United Nations [11] reported the COVID-19 pandemic is now reversing the progress that was registered in health. Before the pandemic, the world made a lot of progress in improving the health of millions of people across the world. Progress was made in improving life expectancy and the reduction of some diseases associated with child and maternal mortality [11,13]. The United Nations [10] also stated that the pandemic provides a watershed moment for health emergency preparedness and investment in critical 21st-century public services. When it comes to the application of AI and machine learning in addressing the SDGs from one up to seventeen, there is significant evidence that shows that, if properly harnessed, AI can help in attaining most of the goals. AI capabilities are currently used to further societal goals; for instance, AI applications combined with satellite imagery were employed in Houston after Hurricane Harvey to identify safe escape routes for the people who were trapped by rising waters. AI-powered object detection is also at the core of the recent applications that are assisting in bringing relief to many visually impaired individuals who live in developing countries mostly. The Microsoft application, which works with a smartphone, uses AI to recognize friends and describe people and specific objects such as currency bills. This stands as a testimony that, if AI can be used appropriately, it can help in furthering societal goals.

## 3. Telemedicine/Telehealth and Goal Three, Ensure Healthy Lives and Promote Wellbeing for All at All Ages

Telemedicine is defined as “the delivery of health care and the exchange of health-care information across distances” [14]. As a result, telemedicine is not a technology or a branch of medicine. Telemedicine is usually characterized by the interaction of a client and the expert in real-time or pre-recorded. The information transmitted can be text, audio, and even video. Craig and Petterson [14] stated that telemedicine has been in use under the conditions of emergencies where there is no alternative, especially in remote environments. It can also be appropriate to be used in situations where it is better compared to conventional services—for instance, teleradiology for rural hospitals. It is believed that telemedicine has been beneficial in improving equity of access to health care, the quality of the care, and even the effectiveness and efficiency of the delivery of the services [14,15]. Hjelm [15] also argued that telemedicine has some advantages, which include effective and efficient access to information, provision of care to people whom it has never been delivered, improved professional education, quality control of screening programmes, and the reduction in healthcare costs. According to Hjelm [15], despite the benefits of telemedicine, there are challenges related to it, which include the breakdown of the relationships between health professionals and patients, challenges related to the quality of healthcare information, among others. However, despite the challenges, telemedicine is more beneficial—especially when more research is done to reduce the challenges. Fagherazzi et al. [3] stated that during the pick of the COVID-19 pandemic telemedicine played a critical role in allowing patients to receive care while at home, avoiding the spread of the virus in waiting rooms. Fagherazzi et al. [3] also argued that telemedicine allowed healthcare professionals to be able to screen, monitoring symptoms in real-time, with the opportunity to give medical advice and to allow stable patients to be able to stay at home to avoid overloaded hospitals. Telemedicine also helped in the collection of meaningful information on patients to assess the evolution of their symptoms in real-time [16] (Portnoy et al., 2020). Telemedicine played an important role in ensuring that people, both doctors and patients, were not exposed to the COVID-19 virus during the pandemic, and apart from the pandemic, studies show that it can be applied to combat future pandemics and effective delivery of healthcare, even on a day-to-day basis, making the objective of ensuring healthy lives and promoting of wellbeing for all at all ages possible. Ohannessian et al. [17] argued that “all stakeholders are encouraged to address the challenges and collaborate to promote the safe and evidence-based use of telemedicine during the current pandemic and future outbreaks”. The other important aspect of telemedicine is that it acts as a reliable source of data. The fact that AI and machine learning depends more on the availability of big data means that telemedicine can play a huge role in the provision of this data. This data can play a diagnostic and preventive role in many diseases, for instance, and it can help to prevent exacerbations. The fact that telemedicine uses information communication technologies to overcome geographical barriers to ensure that healthcare is accessible, even among rural people, makes it possible for big data to be accessed even in remote areas, rural, and underserved communities in developing nations.

## 4. The Fourth Industrial Revolution (4IR)

According to Schwab [18], the 4IR is characterised as “a fusion of technologies that is blurring the lines between the physical, digital, and biological spheres”. The 4IR builds on the developments of the third industrial revolution. To get a clear sense of the industrial revolution, it is very important to understand the first industrial revolution, the second industrial revolution, and the third industrial revolution. The first industrial revolution is well known for the use of water and steam power in the mechanisation of production. In the first industrial revolution, animal power was replaced by the steam engine. In the second industrial revolution, electricity was the main source of energy, which helped a lot in the creation of mass production, while the third industrial revolution used electronics and information technology to automate production. Finally, the 4IR is viewed as the brink of the technological revolution that is changing the way we live, work, and relate to one another. The scale, scope, complexity, and transformation associated with the 4IR is different from all the other revolutions, and it is ushering in experiences that humankind has never experienced before.

As Schwab [18] put it across, the transformations of the 4IR are not just a prolongation of the third industrial revolution, but it is the arrival of a new revolution: the fourth, which is different in velocity, scope, and impact of the system. The other important aspect of why this revolution represents an entirely new revolution is the breakthroughs associated with it, which do not have any historical precedence. Again, the 4IR is evolving at an exponential pace rather than the linear one, which characterised the other revolutions, and it is disrupting almost every industry in all the countries. All the systems of production, management and governance are changing rapidly due to the breadth and depth of the changes associated with this revolution. The 4IR is also characterised by the emergence of technological breakthroughs in AI, robotics, 3D printing, quantum computing, and energy storage, among many other breakthroughs, together with the possibility of billions of people being connected by mobile devices. Schwab [18] went on to indicate that AI is already with us, affecting the various components of human existence—for instance, self-driving vehicles, virtual assistants, and drones, among other countless notable achievements. There has been impressive progress in AI in recent years due to the availability of data and a rise in computing power.

The 4IR also present both opportunities and challenges just as with the revolutions that preceded it. According to Mhlanga, [12] the 4IR can raise global incomes and improve the quality of life for households and individuals in the world. People are benefiting a lot from the opportunities presented by the digital world, ranging from new products and services that improve pleasure and efficiency for ordinary people, such as ordering a cab, fast and efficient ways of purchasing products, convenient ways of listening to music, watching films, and even playing a game, which can be done remotely. There are also supply-side miracles that are being fuelled by technology, which improves efficiency and productivity. Costs related to services such as transportation and communication are dropping because of the application of artificial intelligence. All these improvements will help in the improvement of economic growth as well as the opening of new markets. However, scholars are pointing out that the revolution can lead to greater inequality, especially due to the problem of disrupting the labour market. There are also problems related to major shifts in demand and supply, as well as changes in the way people live and are governed.

Businesses are feeling a shift both on the demand side and the supply side. On the supply side, the introduction of new technology is helping in creating new ways of providing services to customers. The most worrying aspect is that existing industries are being disrupted heavily and, in some instances, the disruption is flowing from agile, innovative competitors who are ousting well-established incumbents at a faster rate through the availability of digital platforms that are enhancing research, development, marketing, sales, and distribution. Research and development, aided by digital technologies, are helping in the improvement of the quality speed and price of the products and services. On the demand side, there are changes experienced in line with transparency, consumer engagement, and new patterns of consumer behaviour, which are premised on access to mobile networks and data. The changes in demand are putting pressure on companies to change the way of design, marketing, and delivering products and services. In short, the 4IR impacts businesses in four major ways, which are customer expectations, product enhancement, collaborative innovation, and organizational forms. The shift from simple digitization of the third industrial revolution to innovation, which is premised on a combination of technologies, in the 4IR is exerting pressure on firms to re-examine the way they do business.

In terms of governance, technology is increasingly changing the way governments across the globe govern. Technology is making governments able to gain technological powers for them to be able to have maximum control over the citizens, using pervasive surveillance systems and the power to have maximum control of digital infrastructure. On the other hand, the convergence of the physical, digital, and biological worlds is allowing citizens to productively engage with the government, present their concerns and opinions, coordinate their efforts, and even circumvent the supervision of public authorities. Technology will allow citizens to exert pressure on governments to change the way they operate. The survival of governments will only depend on their ability to adapt. Aspects of national and international security will also be impacted heavily, and this will have a huge impact on the probability and the nature of the conflict. On people, the 4IR will change not only what people do, but also what it means to be human. The 4IR is affecting what it means to be human, the human identity, the sense of privacy, the notion of ownership, the consumption pattern, the time devoted to leisure, and carrier development, among other notable achievements. However, one of the critical challenges of technology is privacy. It is believed that people will lose control of personal data. Despite all the developments around technology, the good part of it is that humans still have control over it. As Schwab [18] put it across, humans should be responsible for the evolution of technology through the decisions we make daily as citizens, consumers, and investors. There are so many technologies driving the Fourth Industrial Revolution, which include Artificial Intelligence, Big Data, Blockchain, Cloud Computing, Autonomous Vehicles, Quantum computing, Robots and Cobots, and the Internet of Things, among others. These Technologies are clearly outlined in figure one below.

**Figure 1:**
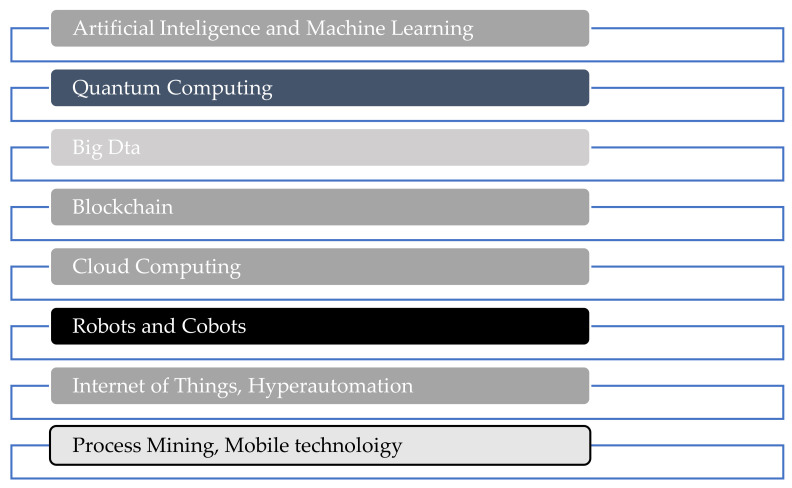
Technologies Driving the Fourth Industrial Revolution. Source: Author’s Analysis.

The technologies listed in Figure 1 above Artificial Intelligence, Big Data, Blockchain, Cloud Computing, Autonomous Vehicles, Quantum computing, Robots and Cobots, and the Internet of Things are driving the Fourth Industrial Revolution. These technologies are being applied in various sectors of the economy including the health sector. D’Alfonso, S. [19] argues that artificial intelligence and machine learning are being applied in mental health to develop prediction, detection, and treatment solutions for mental health care. Again, artificial intelligence is being incorporated into digital interventions, such as web and smartphone applications, to improve user experience and the optimization of personal mental healthcare. All of these point to the fact that AI and machine learning are being applied in the healthcare sector [20]. This fits well with the objective of the current study, which is there to investigate the role of AI and machine learning in addressing the COVID-19 Pandemic and to understand the lessons we are learning on the fourth industrial revolution and the sustainable development goals. Goal three, to be specific, is on good health and well-being. Before explaining the role of AI and the lessons we are learning on the Fourth Industrial Revolution and sustainable development goals, a brief explanation of AI will be given in the next section.

## 5. Brief Description of AI

AI is a broad branch of computer science that create systems that can mimic or function intelligently and independently. In other words, AI is a constellation of various technologies that work together to assist machines to sense, comprehend, act, and learn with a level of intelligence that compares to that of humans. There are two classes of AI: narrow AI, also called weak AI, and general AI, also called strong AI. Narrow AI is what we experience in our everyday lives, AI performs single tasks or a combination of related tasks, such as weather applications, digital assistants, among others. These technologies and algorithms are powerful, but the narrow playing field leads to the word narrow and weak. Weak or narrow AI has massive transformational power, especially if it is applied correctly and has the power to influence how we work and live on a global scale. In a way, weak AI mainly focuses on driving efficiencies in various settings.

On the other hand, general AI, which is also called strong AI, is a form of AI where sentient machines emulate human intelligence, thinking strategically, abstractly, and creatively with the capability to handle a wide range of complex tasks. The full realisation of general AI is yet to come. Currently, AI remains an extension of human capabilities not a replacement of human capabilities. As a result, it is important to have effective human-machine collaboration. AI can come in the form of statistical learning where machines mimic human speech and listen to communicate through language, which is also called the field of speech recognition. Much of speech recognition is statistically based; hence, it is commonly referred to as statistical learning. There is also the field of natural language processing where the machine can write and read a text in a language. The field of computer vision is where machines can see and process information. The field of computer vision is in the category of the symbolic way of computers process information. The other field of AI is pattern recognition where machines can figure out patterns, such as groupings of similar objects. This field requires more data and dimensions of data, and this is the field of machine learning. Then, the field of robotics is where machines can understand the environment and move around fluidly.

Concerning the human brain, the human brain is made of a network of neurons that are used in learning things. Thus, machines use the same structure and function to get cognitive capabilities, which are known as the field of neural networks. The field of deep learning is when the networks are complex and use the networks to learn a complex thing. There are various types of deep learning in machines, which are the different techniques to replicate what the human brain does. A convolution neural network is when the network can scan images from left to right and top to bottom. A convolution neural network is normally used to recognize objects in a scene. This is how computer vision fits in, and object recognition is accomplished through AI. Neural networks can be recurrent where they can remember the limited past, best known as a recurrent neural network. Neural networks can work in two ways: symbolic based and data-based. The database is also known as machine learning, where the machine is fed with lots of data before it can learn. On the other hand, symbolic learning uses symbols to represent certain objects and concepts, and it allows developers to define relationships between them explicitly. We can use all these machine learning techniques to do one of two things: classification or prediction.

## 6. Brief Outline COVID-19 and the Global Economy

The COVID-19 pandemic came with disruptions across all sectors of the global economy. The pandemic affected every sector; even schools and churches were affected due to the dire effects of the pandemic. Companies closed, and others even failed to recover due to the negative effects of the pandemic. Major companies in China, such as Evergrande Group, struggled was heavily affected by the pandemic, and the country even went through an energy crunch, which forced the national government to embark on a serious regulatory crackdown. The prices of fuel and food are rising across the world, and the situation is exacerbated by delays in the movement of goods, due to congestion at ports of entry and strained supply chains, which forces the prices of goods to go up. The other serious problem brought by the pandemic is the shortages in labour in some countries. For instance, in the United Kingdom, it was reported that there was a shortage of truck drivers to transport food from one region to the other, and the country had to introduce temporary visas for 5000 lorry drivers to work in the UK. It was reported that these shortages were caused by a combination of factors, which include COVID, Brexit, and others. The energy shortages in countries such as China have serious problems for production, as manufacturers revised production levels downwards, forcing economists to cut the growth forecasts. The United Nations Department of Economic and Social Affairs [21] indicated that the rapid vaccination rollouts led to an improvement in growth prospects in a few economies. After a sharp contraction of the global economy by 3.6 per cent in 2020, the global economy is projected to expand by 5.4 per cent in the year 2021.

Massive growth in the United States of America and China led to an improvement in prospects for a global recovery, even though the growth prospects could not be enough to lift the rest of the economies of the world. For instance, The United Nations Department of Economic and Social Affairs [21] reported that, despite the growth projects in the world, the economic outlook of countries in South Asia, Sub-Saharan Africa, Latin America, and the Caribbean remains fragile and uncertain in 2021. The pandemic is threatening many vulnerable nations to lose a decade due to insufficient fiscal space to stimulate demand and the risk of a prolonged pandemic. In several developing nations, the economic output is expected to return to pre-pandemic levels in 2022 and 2023. Concerning poverty, The United Nations Department of Economic and Social Affairs [21] reported that the COVID-19 pandemic pushed approximately 114.4 million people into extreme poverty, and among 57.8 million were women and girls. It is reported that women suffered job and income losses more compared to men due to the demands of looking after the children at the height of the pandemic. The fact is that more women, representing many health service workers, caregivers, and essential service providers contributed to the high number of women being pushed into the poverty bracket. The table gives a summary of the growth of the world output and gross domestic product.

Table 2 above is showing the impact of COVID-19 on GDP growth for the whole world and the developed nations. The figures outlined in the table are showing that world output was seriously affected by the COVID-19 pandemic. When compared to 2019 GDP growth, there was a huge decline in world output in 2020 by approximately −3.6. In 2019, world output grew by 2.5. The COVID-19 pandemic seriously affected the world. Concerning developed nations, the growth of GDP was affected drastically, in 2020, to an extent that the growth in GDP fell by 5 per cent. In countries such as the United States of America, Japan, and the United Kingdom, COVID-19 had a huge impact. In the United Kingdom, Great Britain, and Northern Island, there was a 9.9 per cent fall in GDP in 2020, caused by the negative impact of COVID-19. In other developed nations, GDP fell by 3.5 per cent. All this is testimony that the COVID-19 pandemic had a huge impact on world economies. Table 3 is outlining the GDP growth of economies in transition during the COVID-19 pandemic.

In Table 3 above, for economies in transition, COVID-19 had a huge impact on the growth of GDP. For all the economies in transition, GDP fell by 2.7 from a growth of 2.2 in 2019. South-Eastern Europe was also affected by COVID-19, and GDP fell by 3.5 from a growth of 3.7 in 2019. Commonwealth of Independent State and Georgia was also impacted the GDP fell by 2.6 from a growth of 2.2. Russian Federation was not spared, and GDP fell by 3 from a growth of 1.3. The information presented in the table above only shows that the COVID-19 pandemic affected almost every part of the economies in transition. Table 4 summarises the growth, the impact of COVID-19 on GDP for developing nations, and the world trade of goods and services.

Table 4 is giving the growth in GDP for developing countries and the least developing countries. The GDP for developing economies declined by 1.7 in 2020, due to the negative implications of the COVID-19 pandemic. When it comes to Africa, GDP fell by 3.5 from a growth of 2.9. Northern Africa was severely affected, and there was a 5.5 per cent fall in GDP from a 3.2 growth in 2019. Regions of developing nations that were hugely affected were Mexico and Central America, with a decline in GDP of 8.2, followed by the Caribbean with a decline in GDP of 8.1. Southern Africa was also a region that experienced a huge knockdown in GDP, with GDP falling by 6.1. The continent that experienced a growth in GDP was East Asia with a 1 per cent growth in GDP. China is also one of the countries that registered a growth in GDP. GDP grew by 2.3 per cent in China compared to 6.1 growth in 2019. For least developed nations, GDP growth fell by 0.3, which was small compared to other nations in developed nations. In summary, the Pandemic had a huge impact on the growth in GDP for almost all the countries of the world. One other aspect that was affected was the world trade of goods and services, which fell by 8.1 per cent in 2021.

According to the OECD [22], the COVID-19 pandemic exposed vulnerability in the healthcare systems around the globe. The OECD indicated that the pandemic would have serious implications for health, social cohesion, trust in governments, and economic progress. According to the United Nations before the pandemic, there has been progressing in the improvement of the health of a lot of people around the globe. Progress was made in improving life expectancy and the reduction in the common killer diseases associated with child and maternal mortality. The United Nations also admit that more effort is required to ensure that many diseases are fully eradicated. It was highlighted that “the provision of more funding of healthcare systems, improved sanitation and hygiene, and increased access to physicians, significant progress can be made in helping to save the lives of millions”. The pandemic, on the other hand, led to the serious loss of human life, presenting an unprecedented challenge to public health. The World Health Organization [23] reported that, as of 31 December 2020, approximately 82 million people were infected, and more than 1.8 million were killed worldwide. The preliminary estimates from the World Health Organization also pointed to the fact that excess deaths, directly and indirectly, attributable to COVID-19 in 2020 were approximately 3 million, which was 1.2 million higher than the official figures reported by various nations to the World Health Organization. The number of deaths and the people infected seriously affected the health care systems around the globe to an extent that the progress that was registered about life expectancy was reversed by the pandemic. In the next section, a brief review of the literature will be given to try and place the current study in a broader context.

## 7. Brief Review of Literature

Literature documenting the impact of Artificial intelligence on COVID-19 is growing and encouraging. For instance, Senthilraja [6], Adadi et al. [24], and Islam et al. [5], among many other authors, documented how AI has been applied in health, particularly in addressing the impact of COVID-19. However, in the literature reviewed, there is consistency in the application of AI in health, particularly in addressing the negative implications of COVID-19. The current research seeks to investigate the lessons we are learning on the fourth industrial revolution and the sustainable development goals, goal three to be specific, on good health and well-being. Senthilraja [6] found out that AI plays a critical role in combating the negative implications of COVID-19. For instance, Senthilraja [6] found that AI has been used in activity predictions such as physicochemical properties. Senthilraja [6] further found that AI has been helpful in the treatment and health monitoring of COVID-19 patients. AI has been applied in tracking COVID-19 at various scales such as medical, molecular, and epidemiological applications. Senthilraja, [6] also insinuated that AI has been helpful in COVID-19 research by assisting in the analysis of the available data and drug development. All this information by Senthilraja [6] is a testimony that AI has helped address the negative implication of COVID-19.

Another study by Adadi et al. [24] revealed that AI played a huge role in the response to the COVID-19 pandemic. Adadi et al. [24] stated that the growing interest in using AI in handling COVID-19 issues has led to an increase in AI research, leading to a rise in the number of articles and review studies in a short space of time. A study by Islam et al. [5] also highlighted that the application of AI in health has been on the rise, and the rate has been increased exponentially by the COVID-19 pandemic. Yu et al. [25] also alluded to the fact that AI is changing medical practice. One reason given by Yu et al. [25] for the increase in the use of AI in the health sector is the progress that is being registered in big data acquisition, machine learning, and the advances that are being experienced in computing infrastructure development. Yu et al. [13] also argued that AI is now being applied in areas that were previously reserved for humans due to the progress being made in data acquisition and the increase in computing power.

Another study by Davenport and Kalakota, [26] also supported the arguments put forward by Yu et al. [25] (2018). Davenport and Kalakota [26] stated that the rise in data in healthcare is making AI be applied in the healthcare sector. Davenport and Kalakota [26] posit that AI is being applied in treatment recommendation and diagnosis, and in some instances, it is being applied in patient engagement and adherence and even for administrative purposes. Davenport and Kalakota, [26] also pointed out that even though AI can now be applied in many circumstances even in areas previously performed by human beings, some implementation constraints will make it impossible to outright replace humans soon. Reddy et al. [27] also came up with arguments that support the ideas of Yu et al. [25] and Davenport and Kalakota, [26]. Reddy et al. [27] also stated that, in recent years, AI technology has been progressing at an exponential rate with the development of deep neural networks, robotics, computer vision, and natural language processing. Reddy et al. [27] alluded to the fact that all these AI technologies are being applied in healthcare to the extent that, in the coming years, the jobs of clinicians and administrators will likely be taken over by AI. One outstanding argument by Reddy et al. [27] was that, even though AI will play a pivotal role in the delivery of healthcare, it is inaccurate to conclude that AI will take over and replace the roles of human clinicians, but AI will have a meaningful impact on clinical decision support, health interventions, patient monitoring, and patient administration. Reddy et al. [27] believe that AI will play a critical role in AI-enabled or AI-augmented health systems.

Sipior [28] also stated that AI is playing a very important role in fighting COVID-19, and it is contributing quick solutions, which were impossible to be achieved, in various fields and applications. Sipior [28] pointed out that, since the outbreak of COVID-19, there has been an upsurge in the exploration and use of AI, together with many data analysis tools, in various areas. Sipior [28] addressed the management considerations for the successful deployment of AI applications, which include planning, the possibility of biased results, the importance of data, and diversity in AI team membership, among many other considerations. Finally, Sipior [28] concluded that there is a need for careful consideration of issues that are associated with the development and use of AI as humanity look for quick solutions. Vaishya et al. [8] also reviewed the role played by AI in the analysis, prevention, and fight against COVID-19 and other pandemics. Vaishya et al. [8] discovered that AI has been applied in seven different ways, which include the detection of cluster cases and the prediction of where the virus will have a strong effect in the future, through the collection and analysis of data. Vaishya et al. [8] also stated that AI can also play a pivotal role in vaccine development, prediction, and tracking of current patients and future patients. It was also highlighted that AI works very proficiently in mimicking human-like intelligence. In this literature review, AI is doing a lot in addressing the problems related to COVID-19. The question that remains is: what lessons are we learning on the fourth industrial revolution and the sustainable development goals from the application of AI in addressing the effects of COVID-19?

## 8. Methodology

In this study, secondary desktop research was employed to investigate the role of AI in addressing the COVID-19 Pandemic and to come up with lessons we are learning on the fourth industrial revolution and the sustainable development goals—goal three to be specific. Qualitative content analysis was employed in the study. Stemler [29] defined content analysis as “a systematic, replicable technique for compressing many words of text into fewer content categories based on explicit rules of coding”. Prasad [30] also defined content analysis “as the scientific study of the content of the communication”. Prasad [18] went further to argue that “content analysis is the study of the content regarding the meanings, contexts and intentions contained in messages”. Stemler [29] posits that, in the era of big data, content analysis, as a methodological technique, can be one of the powerful tools that researchers can use effectively and efficiently. Stemler [29] believes that content analysis can be applied effectively in various modes of data such as textual, audio data, and visual data. Because there has been a massive explosion of COVID-19 research on various topics, including the role of AI in addressing the negative implications of COVID-19, the technique of content analysis appears to be more effective. Again, the fact that the data on COVID-19 appears in different forms such as photographic, video, audio and text, the use of content analysis proved to be an effective method to use. This is chiefly because the content analysis can be used in various data sources, which can be textual data, visual stimuli such as photographs or video, and even audio data. Stemler [29] also states that the content analysis method is highly flexible as it can be either empirically or theoretically driven.

Table 5 above gives a summary of all the sources consulted in the content analysis. The documents include journal articles from different journals, reports, and media articles, among others.

## 9. Results and Discussion

Since the beginning of the COVID-19 pandemic, the power of AI as one of the Fourth Industrial Revolution technology has been visible to some extent but, at the same time, unmonitored [7]. AI applications have been applied in the health sector for a variety of purposes, which include drug development and approval, monitoring the movement of the population, disease forecasting, among other notable uses. The coming of the pandemic caused a surge in the use of AI applications to address the negative consequences of the virus. The following section will document the areas where AI was applied in combating the negative consequences of the virus. It is important to point out that all the applications of AI and machine learning were possible due to the increase in the availability of Big Data.

### 9.1. Speeding up Research and Treatment of the COVID-19 Related Complications

One area where AI proved to be effective and efficient in the fight against COVID-19 is the development of COVID-19 vaccines. AI performed a critical role in the development of the COVID-19 vaccines and even in the identification of the old vaccines that can be transformed into other new vaccines and therapeutics [7]. The process of developing drugs and vaccines has naturally been long, and it combines different basic science disciplines such as biology, chemistry, and pharmacology [7,31]. Figure 1 below is summarising the traditional way of developing drugs and the timelines involved in the process.

**Figure 2:**
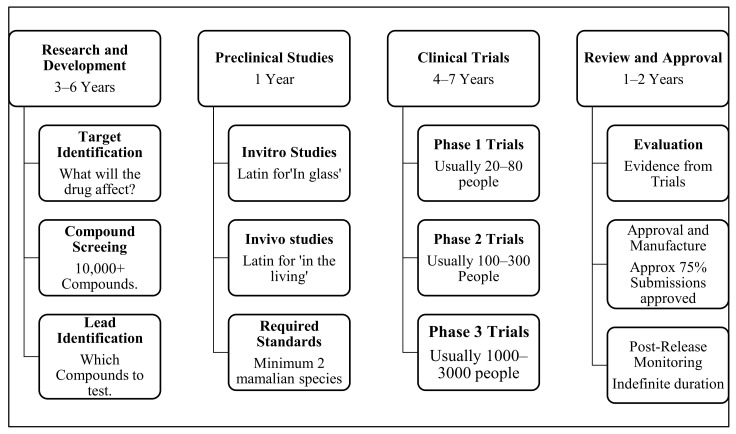
Traditional drug development process. Source: Author’s Analysis Information was adapted from Harrus and Wyndham [19].

In Figure 2 above, the schematic view of the traditional drug development process is shown clearly. The research and development took approximately 3–6 years involving issues related to target identification, compound screening, and lead identification. Preclinical trials took approximately 1 year, with clinical trials taking approximately 4–7 years. Review and approval of drugs normally take 1 to 2 years. According to the FDA [32], out of the hundreds and thousands of chemical compounds that are made and tested to discover the one that meets the requirements, it’s only one in a thousand drugs that usually progresses from preclinical testing to clinical trials. The FDA [32] also alluded to the fact that it is normally one in ten drugs that manage to enter phase 1 of clinical trials that will be developed for sale. DiMasi et al. [33] also revealed that overage takes about 10 years and more than 2.5 billion to successfully develop one drug that will be approved by the US Food and Drug Administration. Considering the existence of the COVID-19 pandemic, pharmaceutical companies had to come up with other innovative ways to develop the COVID-19 vaccines that will help to cut costs and to reduce the time frame to fight the COVID-19 pandemic. Harrus and Wyndham [7] insinuate that AI proved to be one of the Fourth Industrial Revolution factors that could help in the development of drugs with a positive impact on the reduction in time and costs involved. Harrus and Wyndham [7] posit that the power of AI-based algorithms in drug development was realized starting in mid-2010. Various pharmaceutical companies either acquired, merged, or formed some collaborations with AI-centred software companies to realise the benefits of AI in the development of drugs.

Smalley [34] reported that AI-based algorithms can be used in the initial stages of drug development to reduce the number of compounds considered and remove the drugs considered to cause adverse reactions. Harrus and Wyndham [7] argued that the application of AI in drug development and re-purposing of the existing drugs increased because of the COVID-19 pandemic. The re-purposing of existing drugs proved to have the benefit of reducing the time of drug approvals because the drugs are already being used with side effects that are measured and known. The process of approving the drugs will depend on the effectiveness of the drug for use, rather than the purpose it was approved for initially. According to Richardson et al. [35], a start-up called Benevolent AI that uses AI for drug development and identification came forth with the suggestion to use a rheumatoid arthritis drug called Baricitinib to address the severe symptoms of COVID-19. Following the information, the maker of the drug, Eli Lilly, had some partnerships with the US National Institute of Allergy and Infectious diseases, and the clinical trials proved to be effective. Without the power of AI, it was going to be impossible to establish the connection between the arthritis drug and COVID-19 [36,37]. AI-based algorithms proved to be powerful in the development and re-purposing of drugs to fight against the negative implications of the COVID-19 pandemic. Harrus and Wyndham [7] argued that the research on repurposing and developing drugs should grow even after the COVID-19 pandemic. This information on the power of AI, in developing and repurposing drugs, is telling us that the Fourth Industrial Revolution and the technologies driving it such as AI have the potential to contribute immensely to the attainment of sustainable development goals, goal three to be specific.

### 9.2. AI and Forecasting and Scaling Customer Communications during the Pandemic

The first applications to discover the COVID-19 virus was the forecasting applications powered by AI, and the success of the forecasting applications motivated a massive use of AI in the fight against the COVID-19 pandemic. AI applications were widely used for providing insights about the pandemic throughout the whole world. One example is BlueDot a health monitoring company in Canada that alerted its employees and customers about the possibility of an outbreak of new pneumonia-like disease coming from China Wuhan Province. According to Neiiler [38], the information from BlueDot to its customers came 10 days earlier than the warning from the World Health Organisation (WHO) and even seven days earlier than the information from the United States Centers for Disease Control and Prevention (CDC). The use of big data, both health-related and non-health-related, allowed the company to take advantage of AI techniques to forecast the outbreak of the disease and the way the disease would be spreading. One outstanding achievement by BlueDot was to be able to predict, with clarity, the cities where the disease was going to be detected next [38]. Another important use of AI was to predict how COVID-19 spread and the intensity of danger posed by the virus. For instance, Li et al. [39] came forth with a study that analysed the available data on the Hubei epidemic situation. Through the power of big data, Li et al. [39] managed to predict the evolution trend of the virus through the existing data. The research managed to come up with controls that were very useful for the pandemic. Through the existence of data, Li et al. [39] were able to predict the development trends of the pandemic in countries such as South Korea, Iran, and Italy. All this information is a testimony that AI was applied successfully in predicting the spread of the virus to reduce the spread and the negative effects of the virus. Senthilraja [6] stated that, before the virus was even known to the world and its threats, AI systems had already detected its outbreak, and it is important to continue using the various AI applications to support the work of policymakers in the medical community and society, so every stage of the crisis is well managed—even post-pandemic. In a way, the information presented above only shows that AI has a great deal to contribute toward the development of relevant policies that can help to address future pandemics and even sustainable development goals [6]. The other aspect where the application of AI was very useful in the fight against the pandemic was through diagnosis, containment, and monitoring of the virus.

### 9.3. Diagnosis, Containment and Monitoring

One of the equally important aspects of containing the virus was to do with the effective diagnosis of the virus. With the rate at which the virus was spreading, diagnosing the virus and screening it with speed was one of the areas where the success of containing the virus was lying. According to Nguyen [40], various AI applications were proposed during the height of the pandemic. These applications were used differently in different circumstances, and some were not successfully implemented at a large scale, but they were applied at small trials due to difficulties to train efficient and effective AI models, using data that has problems in reflecting the population composition on which the AI models are applied. Despite these problems, the importance of AI applications grew because speed in diagnosing cases of COVID-19 was also saving on hospital bed allocation, among other initiatives. According to Zhou et al. [41], in China, applications that made use of various deep learning techniques grew to greater than 100 as early as March 2020, and in Italy, these applications were applied as early as April 2020, as revealed by [42]. Zhou et al. [41] alluded to the fact that some of the AI applications were useful in separating COVID-19 chest X-rays from other types of diseases such as influenza pneumonia. Kondylakis et al. [43] indicated that several mobile applications were developed to try to combat the negative impact of the COVID-19 pandemic to flatten the curve from the rising numbers of the virus. Kondylakis et al. [43] insinuated that various mobile applications were used for information sharing, self-management of symptoms, contact tracing, making decisions, risk assessment, and home monitoring. Singh, et al. [44] also found out that various mobile health applications were used in mitigating the COVID-19 disease, mostly for contact tracing and symptom monitoring. Again, Patel [33] also alluded to the idea that mobile applications can also be used for remote first-degree triage of people taking a cough test for additional screening and medical attention. Patel [45] argued that most of these mobile applications were important in avoiding unnecessary hospital visits and the overuse of limited medical resources. Senthilraja [6] also went on to posit that AI has the power to track and forecast the nature of the virus from the big data available from platforms such as social media, and even media platforms, with the risk of infection and the rate at which the disease is spreading. The other important aspect is that AI can predict the positive cases and deaths in any region, which is important in preparing mechanisms for fighting the pandemic.

### 9.4. AI and Understanding How COVID-19 Spreads, Treatments and Cures

One of the applications that were used immensely was geofencing or green passports. Before the pandemic, geofencing was used as a marketing tool by monitoring the position of a cellphone to locate the location of the owner. This was done to alert people about nearby stores and products. With the pandemic, geofencing was highly used for quarantine purposes by commercial companies. According to Hui [46], in China, geofencing was implemented to monitor people when they are being quarantined. The other use of geofencing was to mark the infected areas and give information to health authorities. All these were happening due to the power of AI [47]. According to Wesner [48], in the United States of America geofencing was not applied due to problems related to curtailing freedom of movement and the potential to be abused for selfish interests. In Australia, a vaccine passport was introduced where all the people receiving the vaccine are recorded in a centralized database: the Australian Immunization Register, which contains all the people who were immunized. Countries such as Denmark, the European Union, Israel, and the Netherlands all implemented the vaccine passport. Senthilraja [6] also highlighted that AI has been instrumental for diseases such as COVID-19 due to its demands for surveillance. The fact that issues related to human activity, such as migration, were responsible for the spread of the virus around the world means there was a need for applications that can help in monitoring the movement of people and monitor the spread of the virus. One example given earlier is Blue Dot, a company that leveraged the power of AI machine learning and natural language processing to track and report the spread of the virus. Senthilraja [6] also highlighted that AI is useful in the treatment and cure of COVID-19 related illnesses, especially with the help of real-time data analysis. Analysis of data usually provides information that is up to date and will assist in preventing the spread of the disease. AI information can also be used in predicting the sites of infection, the influx of the virus and the information provided by AI can also go a step further to show the need for beds and healthcare [6]. Senthilraja [6] also argued that AI can act as an important tool in the prevention of future viruses by identifying the traits, the causes, and the reasons why the virus is spreading.

### 9.5. AI Lessons on the Fourth Industrial and Sustainable Development Goals

Taking from what AI managed to achieve in a short space of time in the fight against COVID-19, we can also learn a lot about its impact on sustainable development goals, goal three to be specific. The way AI performed in the fight against COVID-19 can also give us some lessons on the implications of the Fourth Industrial Revolution in the attainment of goal three, “Ensure healthy lives and promote well-being for all”. According to the United Nations [49], there has been massive progress in the reduction of “child mortality, improving maternal health, and tackling HIV/AIDS, tuberculosis, malaria, and other diseases” since the creation of the Millennium Development Goals. The United Nations also reported that, in 15 years, the number of people newly infected by HIV year by year has been declining from a high figure of 3.1 million to 2 million. Apart from that, it was reported that approximately 6.2 million lives were saved from malaria. The other achievement is the declining maternal mortality, and it was reported that “since 1990 maternal mortality fell by 45 per cent” and globally preventable child deaths have been declining by over 50 per cent. With all this progress, the United Nations reported that the health emergencies posed by COVID-19 highlighted the disparities in countries to recover from the crisis, and although nations were not all set to attain the sustainable development goals by 2030, their efforts were shuttered by the pandemic. The progress of ensuring “health and well-being for all, including a bold commitment to end the epidemics of AIDS, tuberculosis, malaria and other communicable diseases by 2030” was reversed severely. Despite the threats posed by the pandemic, we have witnessed the power of AI in different settings from speeding up research and treatment of the COVID-19 related complications, forecasting, and scaling customer communications during the pandemic, diagnosis, containment, monitoring, and even increasing an understanding of how COVID-19 spreads, how it is treated, and how it is cured. It is against this background that we believe that, if countries invest in AI, it is possible to exceed pre-COVID development trajectories because of the power AI has in transforming economies. The question that remains is: what are the implications of AI in the attainment of sustainable development with three of the sustainable development goals in mind? Figure 2 below outlines the targets of goal 3 of the sustainable development goals.

**Figure 3:**
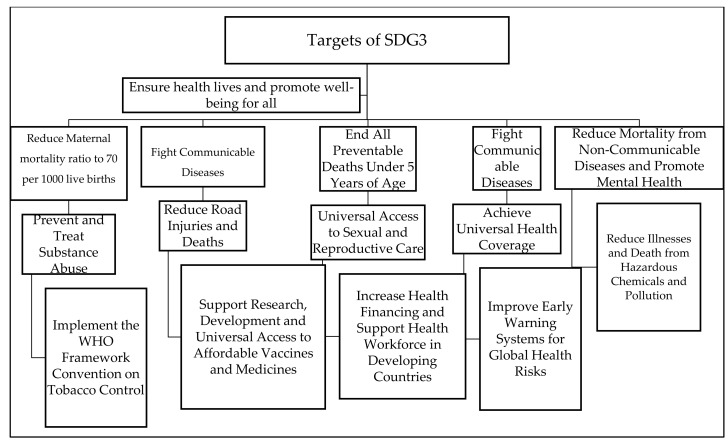
Targets of the Sustainable development goal 3. Source Author’s Analysis.

The United Nations indicated that to meet the global goals, every person can contribute. Taking from what AI has been doing in the fight against the pandemic, we can conclude that AI can help to create the action that can help in promoting health and wellbeing, as well as the attainment of the above targets. AI can help in achieving sustainable development goals in various ways, which include those summarised in Figure 3 below:

The information is presented in Figure 3, and the way AI performed in the fight against COVID-19 is a testimony that, if AI is applied in healthcare, it can go a long way in speeding the attainment of the sustainable development goal 3 and its targets because, with AI healthcare, will be accessible to everyone, as costs will be reduced greatly. This is in line with the arguments by Vinuesa et al. [50], who discovered that the application of AI in different sectors requires serious assessment, especially its impact on sustainable development goals. Vinuesa et al. [50] discovered that AI can help to accomplish 134 targets of the SDGs, and AI development should be supported at the national level, through regulatory insight and oversight for AI-based technologies, to ensure sustainable development is attained. Reddy et al. [51] also supported the idea that AI application in healthcare is becoming increasingly evident, and there is a likelihood that AI will be applied in routine clinical care soon. Reddy et al. [51] also alluded to the fact that the promise that AI is bringing is making governments and technology companies increase their focus on investment in AI medical applications the world over. Holzinger et al. [52] also insinuated that the main driver of digital transformation is AI, and the potential for AI to create benefits for humanity and the environment is undeniably huge. Holzinger et al. [52] believe that AI can assist in finding new ways of solving the critical challenges that face humanity in all areas of our life, from agriculture to healthcare, among many others.

**Figure 4:**
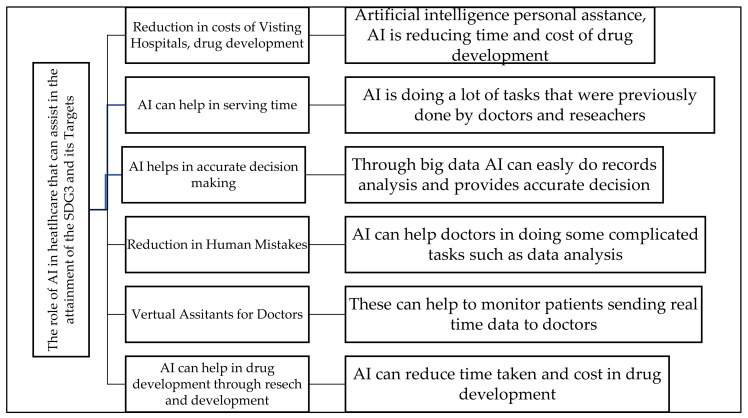
The roles of AI: the role of AI in healthcare can assist in the attainment of SDG3 and its targets in the Post-COVID World. Source Author’s Analysis.

**Figure 5:**
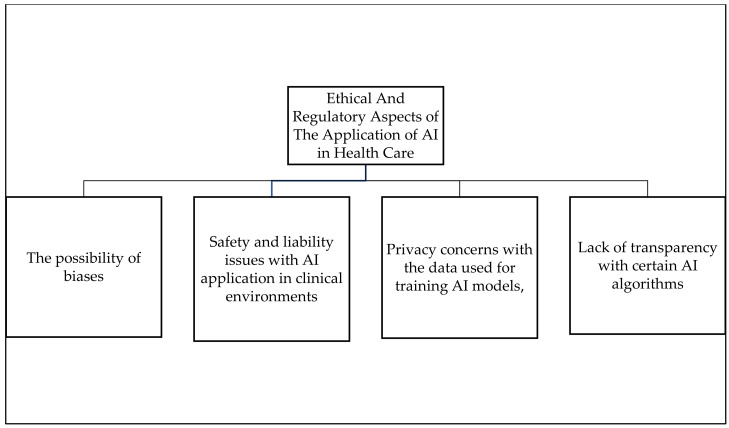
Ethical and Regulatory Aspects of the Application of AI in Health Care. Source: Author’s Analysis.

Despite the power of AI in assisting in the attainment of the targets of goal 3, there are serious concerns raised by authors such as Reddy et al. [51], Morley et al. [53], Truby [54], Holzinger et al. [52], and Mhlanga [55]. For instance, Holzinger et al. [51] state that, even though AI can help to find new solutions to the world’s problems, various unimagined threats are associated with the use of AI, which requires that all stakeholders’ governments, policymakers, industry, and academia ensure that these threats are taken into consideration. Holzinger et al. [51] believe that the “safety, traceability, transparency, explainability, validity, and verifiability of AI applications in our everyday lives are ensured”. Some of the roles AI can assist in the attainment of the SDG3 and its Targets in healthcare are summarised in Figure 4 below:

Figure 4 above is summarising the roles AI can assist in the attainment of SDG3 and its Targets in healthcare. Figure 5 below some of the ethical and regulatory aspects of the application of ai in healthcare.

Figure 5 above is outlining some of the ethical and regulatory issues associated with the use of AI in healthcare. Regardless of these ethical issues, the study discovered that AI contributed a lot towards addressing the problems and risks of the COVID-19 pandemic, which gave us the idea that, if correctly applied, AI and machine learning can help effectively in the attainment of sustainable development goals, with a particular focus in goal 3 and its targets. This can be achieved in many ways, as alluded to before, but more importantly, AI can help to discover disparities in access to healthcare and be used to ensure that healthcare is accessible to everyone.

## 10. Conclusions and Policy Recommendation

The COVID has halted and reversed progress in health and subsequently shortened life expectancy. There has been a rise in interest in research on how artificial intelligence and machine learning handled the COVID-19. This interest led to an increase in the number of review studies and articles targeting the role played by artificial intelligence and machine learning in addressing the COVID-19 pandemic. However, studies that extend to discussion of the fourth industrial revolution and the sustainable development goals were limited. Hence, the current study aimed to assess the role played by artificial intelligence and machine learning in addressing the dangers posed by the COVID-19 pandemic and extrapolate the lessons on the fourth industrial revolution and sustainable development goals. Using qualitative content analysis, the results indicated that artificial intelligence and machine learning played an important role in the response to the challenges posed by the COVID-19 pandemic. Artificial intelligence and machine learning performed meaningful roles in scaling customer communications, provided a platform for understanding how COVID-19 spreads and sped up research and treatment of COVID-19 among other notable achievements. The lessons we draw from this is that, despite the disruptions and the rise in the number of unintended consequences of technology in the fourth industrial revolution, the role played by artificial intelligence and machine learning motivates us to conclude that governments must build trust in artificial intelligence and machine learning, in addressing health problems going forward, to ensure that the sustainable development goals related to good health and wellbeing are achieved. The results of this research are important in creating awareness, among the people, that AI and machine learning, if properly applied, can have a meaningful contribution towards the attainment of the SDGs.

## Figures and Tables

**Table 1:** Sustainable development goals.

Sustainable Development Goals	Description
Goal 1	Put an end to poverty in all of its manifestations around the world.
Goal 2	Put an end to hunger and attain food security enhance sustainable agriculture and boost nutrition.
Goal 3	Ensure that all people of all ages enjoy healthy lives and are happy.
Goal 4	Ensure that all students receive a high-quality education that is inclusive and equitable, and that lifelong learning opportunities are available to all.
Goal 5	Assist all women and girls in achieving gender equality.
Goal 6	Ensure that all people of all ages enjoy healthy lives and are happy.
Goal 7	Ensure that everyone has access to energy that is affordable, reliable, sustainable, and modern.
Goal 8	Encourage long-term, inclusive, and sustainable economic growth, as well as full and productive employment and decent work for everyone.
Goal 9	Build a more resilient infrastructure, encourage inclusive and sustainable industrialization, and encourage innovation.
Goal 10	Reduce intra- and inter-country inequity.
Goal 11	Ensure that cities and human settlements be inclusive, safe, resilient, and long-lasting.
Goal 12	Ensure that consumption and production trends are sustainable.
Goal 13	Take immediate action to address climate change and its consequences.
Goal 14	For sustainable development, conserve and sustainably utilise the oceans, seas, and marine resources.
Goal 15	Defend, rebuild, and promote sustainable use of terrestrial ecosystems, manage forests sustainably, prevent desertification, halt and reverse land degradation, and stop biodiversity loss.
Goal 16	To ensure long-term development, promote peaceful and inclusive societies, ensure universal access to justice, and construct effective, responsible, and inclusive institutions at all levels.
Goal 17	Strengthen and revive the Global Partnership for Sustainable Development’s implementation mechanisms.

Source: Author’s Analysis.

**Table 2:** Growth of world output and Developed Nations.

	2019 GDP Growth	2020 GDP Growth	2021 Projected Growth	2022 Projected Growth
World Output	2.5	−3.6	5.4	4.1
Developed Economies	1.7	−5.0	5.0	3.4
United States of America	2.2	−3.5	6.2	3.2
Japan	0.3	−4.8	3.3	2.2
The United Kingdom Great Britain and Northern Island	1.3	−9.9	5.1	5.5
Other developed Nations	1.7	−3.5	3.6	2.9

Source: Author’s Analysis of the United Nations Department of Economic and Social Affairs data.

**Table 3:** Growth of GDP of Economies in Transition.

	2019 GDP Growth	2020 GDP Growth	2021 Projected Growth	2022 Projected Growth
Economies in Transition	2.2	−2.7	3.3	3.3
South-Eastern Europe	3.7	−3.5	4.2	3.5
Commonwealth of the Independent States and Georgia	2.2	−2.6	3.3	3.3
Russia Federation	1.3	−3.0	3.0	3.0

Source: Author’s Analysis of the United Nations Department of Economic and Social Affairs data.

**Table 4:** Growth of gross domestic product of Developing Economies.

	2019 GDP Growth	2020 GDP Growth	2021 Projected Growth	2022 Projected Growth
Developing Economies	3.6	−1.7	6.1	5.0
Africa	2.9	−3.5	3.6	3.7
Northern Africa	3.2	−5.5	5.6	4.0
East Africa	6.5	0.1	3.3	4.5
Central Africa	1.9	−1.8	3.0	3.2
West Africa	3.3	−1.0	2.7	3.6
Southern Africa	−0.2	−6.1	2.2	2.6
East and South Asia	4.9	−0.1	7.1	5.7
East Asia	5.3	1.0	7.1	5.2
China	6.1	2.3	8.2	5.8
South Asia	3.1	−5.6	6.9	8.3
India	4.6	−6.8	7.5	10.1
Western Asia	1.2	−3.2	3.7	3.4
Latin America and the Caribbean	−0.3	−7.3	4.3	3.3
South America	−0.7	−6.8	4.1	3.1
Brazil	1.4	−4.1	3.0	2.4
Mexico and Central America	0.6	−8.2	4.7	3.6
Caribbean	0.5	−8.1	4.3	6.8
Least developed countries	4.9	−0.3	4.0	5.0
World trade of Goods and Services	1.2	−8.1	9.4	5.7

Source: Author’s Analysis of the United Nations Department of Economic and Social Affairs data.

**Table 5:** Sources that helped in shaping the trajectory of the study.

Journal Articles	Reports	Media Articles	Others
100	25	25	55
Journals targeted were those published from the year 2000 up wards though Work from previous years was considered. Publishers-Springer Nature, Multidisciplinary Publishing, Es, Elsevier Institute of Electrical and Electronics Engineers, etc.	United Nations, The World Bank, The World Health Organization Organisation for Economic Co-operation and Development (OECD) among others	Media articles from Various countries were used for instance United State of America, South Africa, the United Kingdom among other nations.	Various other documents were consulted to come up with the ideas that shaped the trajectory of the study.

Source: Author’s Analysis.
